# Serum Metabolic Characterization of Vitamin E Deficiency in *Holstein* Cows during the Transition Period Based on Proton Nuclear Magnetic Resonance Spectroscopy

**DOI:** 10.3390/ani13182957

**Published:** 2023-09-19

**Authors:** Yuxi Song, Hongyu Wang, Rui Sun, Jinshui Chang, Jipeng Tang, Yunlong Bai, Cheng Xia

**Affiliations:** College of Animal Science and Veterinary Medicine, Heilongjiang Bayi Agricultural University, Daqing 163319, China; syxalz@163.com (Y.S.); why20220827@163.com (H.W.); a13936697304@163.com (R.S.); cjs19980225@163.com (J.C.); tjp20000118@163.com (J.T.); bai53626077@126.com (Y.B.)

**Keywords:** dairy cows, transition period, vitamin E deficiency, serum, ^1^H nuclear magnetic resonance, metabolomics

## Abstract

**Simple Summary:**

The present study is the first to characterize metabolic changes associated with vitamin E deficiency in *Holstein* cows during the transition period. Our results highlight that cows with vitamin E deficiency are more likely to experience a negative energy balance characterized by alterations of common systemic metabolic processes and develop oxidative stress, inflammation, and ultimately liver injury. This study provides comprehensive information on blood metabolic characterization of vitamin E deficiency in dairy cows after calving.

**Abstract:**

Vitamin E, a potent antioxidant, is a necessary and complex micronutrient for cows. During the transition period, vitamin E deficiency (VED) is among the highest prevalent micronutrient deficits in dairy cows. It may eventually result in oxidative stress and immunological malfunction, and it increases the risk of peripartum disorders. At present, detailed data on blood metabolites in VED cows are limited. Consequently, the purpose of this research was to examine the alterations in the serum metabolic profile of VED cows throughout the early postpartum period. Using comprehensive ^1^H nuclear magnetic resonance (^1^H NMR), the alterations in serum metabolic activities of VED cows were analyzed. In total, 28 multiparous *Holstein* cows were assigned according to serum α-tocopherol (α-Toc) concentrations into normal (α-Toc ≥ 4 μg/mL, *n* = 14) and VED (α-Toc < 3 μg/mL, *n* = 14) groups at 21 days postpartum, and their blood samples were collected for biochemical and ^1^H NMR analyses. A *t*-test on independent samples as well as multivariate statistics were used to assess the findings. In comparison with normal cows, VED cows showed significantly worse body condition scores, milk yield, and dry matter intake (*p* < 0.05). Significantly higher levels of serum non-esterified fatty acids, aspartate aminotransferase, low-density lipoprotein, and malonaldehyde were found in VED-affected cows, as well as lesser concentrations of serum albumin, high-density lipoprotein, and total antioxidant capacity in comparison with normal cows (*p* < 0.01), while other vitamins and minerals concentrations showed no distinction between the groups (*p* > 0.05). Furthermore, 24 upregulated serum metabolites were identified under VED conditions. The metabolomics pathway analysis of these metabolites demonstrated that a global metabolic response to VED in cows was represented by changes in 11 metabolic pathways, comprising energy, carbohydrate, and amino acid metabolism. From these results, we conclude that VED cows were more likely to experience a negative energy balance characterized by alterations of common systemic metabolic processes and develop oxidative stress, inflammation, and ultimately liver injury. This study provides the first evidence of metabolic changes in cows with VED.

## 1. Introduction

Vitamin E deficiency (VED) is the most prevalent micronutrient deficiency in dairy cattle, with a herd incidence rate of up to 21% during early lactation [1]. It leads to severe degenerating disorders, a reduction in the immune reaction, and possibly lameness, retained placenta, metritis, and mastitis, thereby bringing substantial financial loss to the global dairy sector [2,3]. Vitamin E, a potent antioxidant, is a necessary and complex micronutrient for cattle [4,5]. Its predominant component in the circulatory system is α-tocopherol (α-Toc) [6]. Vitamin E shortage can be induced by various factors such as alterations in α-Toc intake, elevated oxidative stress and lipid peroxidation, and the transfer of α-Toc into colostrum near calving [3]. To date, the physiological causes or processes underpinning VED during the transition period of high-yielding dairy cows remain unknown.

The plasma/serum levels of α-Toc in high-yielding dairy cattle reduce steadily during prepartum, beginning multiple weeks before calving, achieving a minimum at parturition, staying at low rates during the puerperium time (approximately 3–7 days), and rising afterward [7,8,9,10,11]. According to the National Research Council (NRC, 2001) standards [12], plasma/serum concentrations of α-Toc must be greater than 3 μg/mL throughout the transition period; otherwise, an α-Toc shortage, also known as VED, can occur. Therefore, VED for adult dairy cows is defined as serum α-Toc concentration < 3 μg/mL [1,3].

Over the past three decades, numerous studies have been conducted on the disease risk of VED and the influence of α-Toc supplements on the health of transition dairy cows and heifers. A comprehensive study by Haga et al. [3] revealed that VED in the transition period is a risk factor for peripartum disorders and reduced reproduction in dairy cows and that α-Toc supplements are an excellent method for preventing peripartum disorder in high-yielding dairy cows. Some proteins and metabolites may exhibit alterations during disease initiation and remain at the same altered level throughout the course of the disease. Unfortunately, few studies have investigated the altered proteins and metabolites in cows with VED during the transition period. Only recently, the proteomics characteristics of VED cows during early lactation were characterized in plasma [13]. However, no studies have reported metabolic changes in VED cows until now.

Recently, proton nuclear magnetic resonance (^1^H NMR) technology has been increasingly used for cows to probe the differences in metabolites between diseased and non-diseased states. However, these studies involved negative energy balance [14], inactive ovaries [15], displaced abomasum [16], milk fever [17], mastitis [18], and ketosis [19,20,21,22,23], but not VED. For metabolic phenotyping, ^1^H NMR spectroscopy is among the preferred analytical techniques [24]. The advantages of ^1^H NMR include that the sample pre-treatment is straightforward and non-destructive, and the identification of the metabolite is extensive [25]. Therefore, ^1^H NMR spectroscopy is a reasonable method to obtain more precise and detailed information on cows with VED. Oxidative stress is the consequence of imbalanced redox homeostasis due to a lack of sufficient antioxidants that neutralize reactive oxygen species, whereas metabolic alterations are likely to be mediated by oxidative stress [26]. Given that vitamin E is a strong antioxidant and its deficiency can lead to oxidative stress, we hypothesized that VED changes the metabolic status of dairy cows during the transition period. Thus, this study aimed to test this hypothesis by applying ^1^H NMR spectroscopy to analyze the metabolome of serum in VED and normal cows.

## 2. Materials and Methods

All animal operations were approved by the Institutional Animal Care and Use Committee of Heilongjiang Bayi Agricultural University (Daqing, China) (protocol code DWKJXY2023065; approval date: 15 January 2023).

### 2.1. Animals and Diets

The present study was conducted during the winter (January–February) of 2023. Twenty-eight clinically healthy multiparous *Holstein* dairy cows (singletons, 3.19 ± 0.27 of age, 2.2 ± 0.48 of parity, 635 ± 22.9 kg of weight, 34.6 ± 5.4 kg of daily milk yield, mean ± SD) from an intensive dairy farm of Heilongjiang in China were selected at 21 days postpartum in this research. Cows were assigned into normal (α-Toc ≥ 4 μg/mL, *n* = 14) and VED (α-Toc < 3 μg/mL, *n* = 14) groups based on serum α-Toc concentrations. Cows were kept in separated tie-stall barns bedded with kiln-dried sawdust and fed individually an early-lactation total mixed rations (TMR) diet following parturition. The formulation of TMR for early lactation followed the NRC (2001) [12] standards (Table 1). Cows were fed three times daily and were milked three times daily at 06:00, 14:00, and 22:00. Age, parity, weight, milk yield, and dry matter intake (DMI) were recorded using the specific software (Afifarm, Afimilk, Kibbutz Afikim, 1514800, Israel). Two qualified field veterinarians determined the body condition score (BCS) using a 5-point scale ranging from 1 to 5 with 0.25-unit intervals [27]. Blood samples for biochemical and ^1^H NMR analysis were obtained on day 21 relative to the calving date.

### 2.2. Blood Collection 

Blood samples were collected only once from two groups of cows via the tail vein utilizing vacuum blood collecting tubes without any additives, and kept at room temperature for 30 min, then centrifuged at 3500× *g* for 10 min to obtain serum. Tubes of serum were kept frozen at −80 °C for the biochemical and ^1^H NMR testing.

### 2.3. Serum Detection

Serum levels of vitamin E (α-Toc), vitamin A (retinol), and vitamin C (ascorbic acid) were determined using high-performance liquid chromatography according to Siomek et al. [28]. Serum selenium, zinc, and copper levels were determined by inductively coupled plasma mass spectrometry as previously described [29].

Serum levels of β-hydroxybutyrate (BHB), non-esterified fatty acids (NEFA), glucose, aspartate aminotransferase (AST), alanine aminotransferase (ALT), albumin, total cholesterol (TC), low-density lipoprotein (LDL), high-density lipoprotein (HDL), calcium, phosphorus, and magnesium were analyzed using commercial biochemical assay kits (Mindray Biomedical Electronics Co., Ltd., Shenzhen, China) by the Mindray BS-830S fully automatic biochemistry analyzer (Mindray Biomedical Electronics Co., Ltd., Shenzhen, China) at the Biotechnology Centre of Heilongjiang Bayi Agricultural University. All measurements were conducted following the manufacturer’s guidelines.

The functions of total antioxidant capacity (TAC), superoxide dismutase (SOD), glutathione peroxidase (GSH-Px), catalase, and malondialdehyde (MDA) in serum were measured using commercial assay kits (Nanjing Built Biology, Nanjing, China) following the manufacturer’s instructions.

### 2.4. Sample Preparation

The testing procedure for ^1^H-NMR was carried out as described in [30]. In brief, 28 freezing serum samples were quickly defrosted in the room atmosphere. The serum sample (200 μL) was transferred utilizing a pipette into a 1.5 mL Eppendorf tube, and 400 μL of buffer (45 mM NaH_2_PO_4_/K_2_HPO_4_; 0.9% NaCl; pH: 7.4; 50% D_2_O) was then supplied. Following vortex and centrifugation (16,099× *g*, 10 min, 4 °C), 550 μL of the supernatant was transmitted to a 5 mm NMR tube for additional examination.

### 2.5. NMR Measurements

Proton NMR spectra were acquired on a Varian-600 MHz NMR spectrometer (Varian, Palo Alto, CA, USA) operating at 599.93 MHz. A water-suppressed Carr-Purcell-Meiboom-Gill sequence was used to obtain transversal relaxation periods. In total, 64 transients were captured using a spectral width of 12,000 Hz, a relaxing latency of 2.0 s, a combining time of 100 ms, and an acquisition time of 1.5 s at 25 °C. Reactant concentrations were in the micromolar range due to the detection limit of the NMR system.

### 2.6. Data Preprocessing

The NMR spectra were analyzed utilizing the MestReNova program (version 12.0.0, Mestrelab Research, St. Louis, MO, USA). The free induction decay signal of all the recorded ^1^H NMR spectra was submitted to an exponential window function with a broadening factor of 1 Hz. Then, a Fourier transform was applied to enhance the signal-to-noise proportion, and the spectral stage and baseline correction were conducted manually. The normalization of multivariable statistics followed the normalization of integrated statistics.

### 2.7. Multivariate Statistical Analysis

A multivariate study was conducted on the normalized information using the SIMCA tool (version 15.0, MKS Data Analytics Solutions, Umea, Sweden). First, the ^1^H NMR spectra were subjected to a principal component analysis (PCA) according to the mean-center scale to reveal broad variations. The spectra were subsequently evaluated using the supervised approach of orthogonal partial least-squares discriminant analysis (OPLS-DA). Using the goodness-of-fit parameter (R^2^) and goodness-of-prediction parameter (Q^2^), the performance for every model was detected. The OPLS-DA was employed to assess the statistical significance of changes in metabolite levels and the relevant correlation coefficients.

### 2.8. The Differential Metabolites Identification and the Analysis of the Metabolic Pathway

Based on the number of samples in the present test, the actual level of the correlation coefficient (*r*) > 0.5324 was utilized as the cut-off level for the significance of differential metabolites. The Euclidean distance matrix for the mathematical calculations of the differential metabolites was constructed for every series of analyses. Using the entire linkage technique, the divergent metabolites were clustered and shown as a heatmap of hierarchical clustering. The Kyoto Encyclopedia of Genes and Genomes (KEGG) database (www.kegg.jp/kegg/pathway.html, accessed on 16 July 2023) was used for the differential metabolites linked to several distinct metabolic pathways. The outcomes of the metabolic pathway investigation were displayed using a bubble plot.

### 2.9. Statistical Analysis 

Cows served as the experimental units. Statistical analysis was performed with SPSS 22.0 software (IBM Corp, Armonk, NY, USA). The independent samples *t*-test was used to examine variations in clinical variables (age, parity, BCS, milk yield, DMI, vitamins, and minerals) and biochemical variables between cows with VED and normal cows. Probability (*p*) values < 0.05 and <0.01 were deemed to be statistically significant and extremely significant, respectively.

## 3. Results

### 3.1. Background Characteristics 

Table 2 shows the background characteristics of the VED and normal groups. Age and parity were similar in both groups (*p* > 0.05), while BCS, milk yield, and DMI were significantly lower in the VED group contrasted with the normal group (*p* < 0.05). Serum vitamin E levels were significantly lower in the VED group than those in the normal group (2.59 ± 0.77 vs. 7.33 ± 1.38 μg/mL, *p* < 0.01). No significant differences were observed in other vitamins and minerals levels between the two groups (*p* > 0.05).

### 3.2. Serum Biochemical Measurements

Table 3 shows the serum biochemical indicators of the VED and normal groups. In contrast with the normal group, cows with VED had higher concentrations of serum NEFA, AST, LDL, and MDA and lower concentrations of serum albumin, HDL, and TAC (*p* < 0.01).

### 3.3. H NMR Spectra

**Figure 1:**
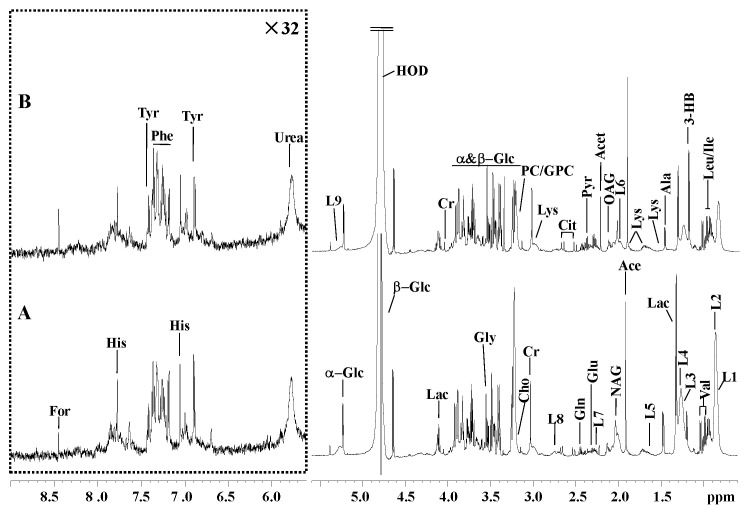
Typical ^1^H-NMR spectra (600.13 MHz) of serum acquired from the (**A**) normal and (**B**) vitamin E deficiency (VED) groups. The horizontal scale represents the chemical shift (ppm). The area with a range of δ5.601–9.0 ppm is magnified 32-fold in comparison to the area with a range of δ0.6–5.6 ppm. For, formate; His, histidine; Phe, phenylalanine; Tyr, tyrosine; L9, lipid, -CH=CH-; α-Glc, α-glucose; Lac, lactate; Cr, creatinine; β-Glc, β-glucose; Ala, alanine; Val, valine; Gly, glycine; PC/GPC, phosphorylcholine/glycerophosphocholine; Cho, choline; Lys, lysine; L8, lipid, =CH-CH_2_-CH=; Dim, dimethylamine; Cit, citrate; Gln, glutamine; Pyr, pyruvate; Glu, glutamate; Acet, acetone; L7, lipid, -CH_2_-C=O; OAG, O-acetyl-glycoprotein; NAG, N-acetyl-glycoprotein; L6, lipid, -CH_2_-CH=CH-; Ace, acetate; L5, VLDL, -CH_2_-CH_2_-C=O; L3, lipid, CH_3_-(CH_2_)n-(LDL); 3-HB, 3-hydroxybutyrate; Ile, isoleucine; Leu, leucine; L2, lipid, CH_3_-(CH_2_)n-(VLDL); L1, lipid, CH_3_-(CH_2_)n-(LDL).

A standard ^1^H NMR spectra of serum samples from the (A) normal and (B) VED groups are shown in Figure 1. All NMR signals (δ) were observed at 0.6–9.0 ppm (ppm represents the chemical shift). Downfield areas were vertically enlarged 32-fold for more clarification. After further excluding the chemical shift range of δ5.601–6.4 ppm affected by the residual water peak, 34 metabolites were identified from the NMR spectra (Supplementary Material Table S1).

### 3.4. Multivariate Statistical Analysis

**Figure 2:**
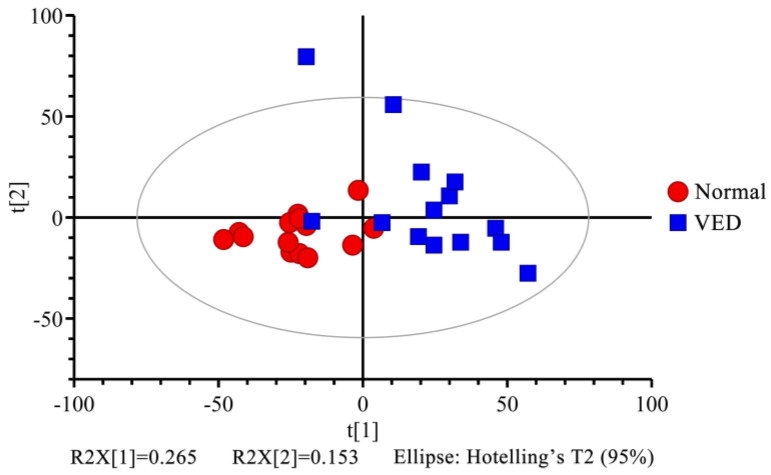
Principal component testing scores of the normal and VED groups plotted side by side. t [1] = initial main component. t [2] = second main component.

**Figure 3:**
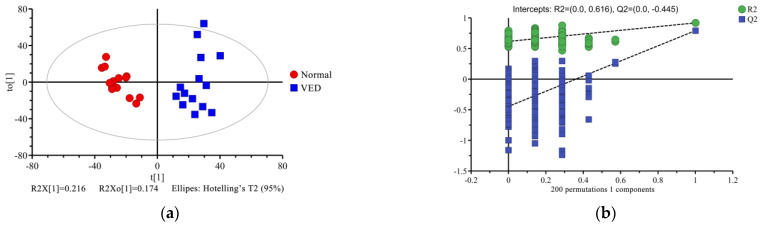
Orthogonal partial least-squares discriminant analysis (OPLS-DA) scores (**a**) and permutation testing of the OPLS-DA framework (**b**) for the normal and VED groups, respectively. t [1] = initial primary element of the matrix. to [1] = orthogonal primary element. The plot of Q^2^ derived from the permutation testing included in the OPLS-DA framework is the intercept limit of Q^2^, which is calculated with the regression line.

To explore the metabolic variations in serum across the normal and VED groups, the ^1^H NMR data were analyzed using multivariate statistics. First, we analyzed the metabolomic profiling of the 34 metabolites with unsupervised PCA. The PCA score plot showed little separation between normal and VED groups (R^2^X = 0.606, Q^2^ = 0.312) (Figure 2 and Table S2). Additionally, an OPLS-DA supervised model was conducted to achieve the best possible distinction (R^2^X = 0.390, R^2^Y = 0.918, and Q^2^ = 0.792) (Table S3). Since the R^2^Y and Q^2^ values were both higher than 0.4, the model was reliable and consistent (Figure 3a). When the Q^2^ result of the OPLS-DA model was close to 1, it achieved a high degree of accuracy. Because the results of the Q^2^ intercept were lower than 0.05, there was no overfitting (Figure 3b).

### 3.5. Differential Metabolites between the VED and Normal Groups

**Figure 4:**
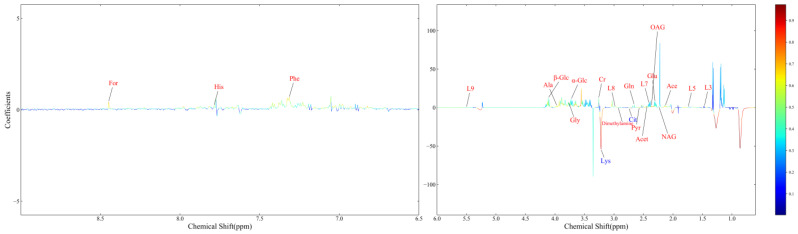
The OPLS-DA loading plot for the normal group and VED group.

**Figure 5:**
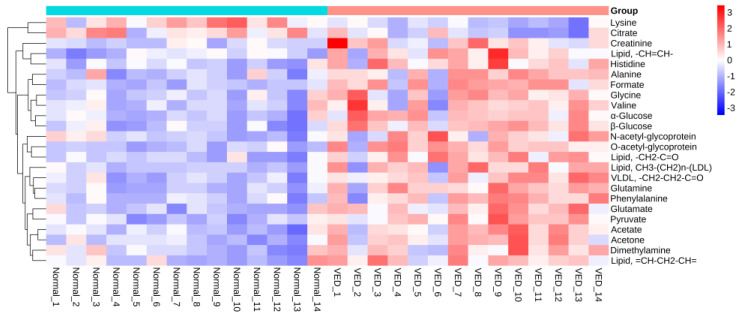
Heatmap visualization of the 24 differential metabolites identified in serum samples from the VED and normal groups.

A polychromatic correlation coefficient loading graph was created (Figure 4) and employed to determine the distinct metabolites that were present in each of the two groups. The absolute value of the correlation coefficient was used as the encoding approach to determine the color of the polychromatic loading graphic. When the color was warmer, the absolute value of the correlation coefficient was higher, and the influence of the color on the intergroup variation was stronger. It was decided that a score of 0.5324 would constitute the cut-off for the overall significance of the correlation coefficient. There was a significant contribution to the intergroup variation created by the parameters matching the correlation coefficient that had an overall value of more than 0.5324 (*p* < 0.05). Table 4 shows the findings of the correlation coefficient assessment of 24 differential metabolites. The VED group, when contrasted with the normal group, had significantly higher serum levels of acetate, acetone, alanine, citrate, creatinine, dimethylamine, formate, glutamate, glutamine, glycine, histidine, LDL, very low-density lipoproteins (VLDL), lipids, lysine, N-acetyl-glycoprotein, O-acetyl-glycoprotein, phenylalanine, pyruvate, valine, and glucose. Furthermore, the relative levels of 24 differential metabolites in the normal and VED groups were visualized on a heat map (Figure 5).

### 3.6. Characterization and Functional Analysis of the Key Metabolic Pathways

**Figure 6:**
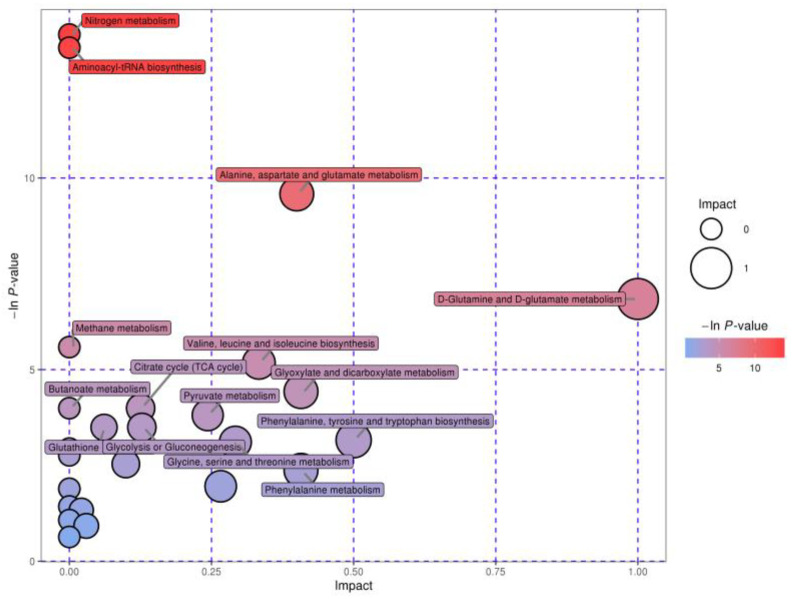
KEGG pathway enrichment analysis comparing the VED and normal groups. Each bubble in the diagram depicts a metabolic pathway, and its abscissa and size show the degree of the pathway influence variables in the topological analyses. The ordinates and colors of the bubbles show the *p* values (−ln *p*-value) of the enrichment test, with darker colors indicating a greater level of enrichment.

To further understand the biological implication of the differential metabolites, we performed metabolic pathway enrichment analysis using MetaboAnalyst 5.0 (https://www.metaboanalyst.ca/, accessed on 16 July 2023). An interactive visualization approach was utilized to illustrate the main pathways for the 24 metabolites that were found to be different between normal and VED cows (Figure 6). The findings of the primary pathway assessment are presented in Table 5. A total of nine pathway impacts >0.1 with *p* < 0.05 were found for the primary metabolic pathways. Among these pathways, four biological modules were involved in carbohydrate metabolism, including glyoxylate and dicarboxylate metabolism (impact = 0.41), pyruvate metabolism (impact = 0.18), glycolysis or gluconeogenesis (impact = 0.13), and citrate cycle (TCA cycle) (impact = 0.13). Five biological modules were involved in amino acid metabolism, including D-glutamine and D-glutamate metabolism (impact = 1.00); phenylalanine, tyrosine, and tryptophan biosynthesis (impact = 0.50); alanine, aspartate, and glutamate metabolism (impact = 0.40); valine, leucine, and isoleucine biosynthesis (impact = 0.33); and glycine, serine, and threonine metabolism (impact = 0.29). Furthermore, nitrogen metabolism and aminoacyl-tRNA biosynthesis were deemed to be possible target pathways (−ln *p* ≥ 10). According to these findings, 11 different metabolic pathways were significantly affected in the cows with VED.

## 4. Discussion

The present study investigated the metabolic changes associated with VED in dairy cows after calving by applying an untargeted metabolomics screening. Following the global non-target metabolomics screening, 24 differential metabolites that can relate to VED were described, and their correlating metabolomic pathways were also identified. The data presented provide the most comprehensive metabolomics analysis of VED in cows to date. These data may support a novel approach for the diagnosis of cows with VED in clinical practice in the future.

Most dairy cattle, during early lactation, undergo a period of negative energy balance (NEB) associated with multiple metabolic changes when DMI is reduced to levels lower than the required energy to maintain the body condition and the production of milk [16,31]. In the present study, we observed lower DMI and milk yield in the VED group relative to the normal group, indicating that VED may be accompanied by NEB. Moreover, the low milk yield can be attributed to a low DMI [32]; the latter may be one of the reasons for VED [7]. In the early phase after parturition in dairy cattle, negative energy balance is typically characterized by substantial mobilization of bodily energy stores [33]. Consequently, the lower postpartum BCS indicated that VED cows suffered an NEB [34]. Increased systemic concentrations of NEFA result from the breakdown of fat storage [35]. This reinforces the circulating NEFA outcomes obtained in the VED group. These data imply that VED cows exhibited an increase in lipid mobilization in the early lactation stage due to NEB.

Abnormal lipid metabolism is associated with the occurrence of VED. Very low-density lipoproteins such as TG-rich lipoprotein are synthesized in the liver [36]. The liver exports vitamin E to extrahepatic tissues through the secretion of VLDL [37]. In the circulatory system, VLDL is transformed into LDL, which becomes the primary transporter of vitamin E isomers to peripheral tissues [38]. Consequently, elevated VLDL values likewise elevate LDL levels [39]. In this investigation, we discovered that circulating VLDL and LDL levels are elevated in VED cows compared with normal cows. This suggests an increased vitamin E consumption in peripheral tissues of VED cows, contributing to the decreased vitamin E in circulation, eventually resulting in VED. In addition, the higher circulating concentrations of unsaturated lipids (L7, L8, L9) in VED cows may potentially be accounted for by the changes in liver fat metabolism [40].

When the energy demand increases but the supply is insufficient, energy metabolism becomes imbalanced [41]. In the current investigation, there was a statistically significant elevation in the serum concentrations of citrate, glucose, and pyruvate when VED was existent. This revealed that the VED cows had an imbalance in their energy metabolism. Citric acid levels, which are a key TCA intermediate, elevated significantly in the serum of the VED group, which indicated that the primary energy supply route, which is the TCA cycle, was blocked. Because the TCA cycle is the main critical system in the energy metabolism of the body, any disruption to this process will eventually result in an energy metabolism imbalance [42,43]. In addition, increased serum glucose and pyruvate levels in VED cows imply promoted gluconeogenesis and altered carbohydrate and energy metabolism. Insufficient energy, in turn, enhances other energy production pathways, such as the creatine and phosphate creatine balance system [44]. Creatine plays a key role in energy metabolism, and the majority of creatine is stored in skeletal muscle as creatine phosphate [45]. Creatinine is a breakdown product of the intracellular creatinine precursors creatine and creatine phosphate [46]. In this study, VED cows exhibited an increase in serum creatinine levels compared to normal cows. This result suggests that NEB in VED cows could be relieved by increasing muscle breakdown.

Vitamin E deficiency can alter amino acid metabolism. Eight differential amino acids were identified in this study. In VED cows, these amino acids were mainly involved in D-glutamine and D-glutamate metabolism; phenylalanine, tyrosine, and tryptophan biosynthesis; alanine, aspartate, and glutamate metabolism; valine, leucine, and isoleucine biosynthesis; and glycine, serine, and threonine metabolism. As the D-glutamine and D-glutamate metabolism pathway had an effective value of 1 showed that the D-glutamine and D-glutamate metabolism pathway as well as the metabolite D-glutamic acid, are major aspects of alterations in amino acid metabolism in VED cows. According to the outcome of their breakdown byproducts, the eight amino acids are classified as either glucogenic amino acids (alanine, glutamate, glutamine, glycine, histidine, and valine), ketogenic amino acids (lysine), or both ketogenic and glucogenic amino acids (phenylalanine) [47]. Gluconeogenic amino acids are those amino acids that when broken down, create either pyruvate or one of the metabolites of the TCA cycle (such as α-ketoglutarate, succinyl-CoA, fumarate, or oxaloacetate), all of which can transform into glucose [48]. On the other hand, ketogenic amino acids can be transformed into ketone bodies through the decomposition of lipids and the production of a source of energy [49]. In the present study, the above glucogenic and/or ketogenic amino acids in the serum were all increased in the VED cows compared with the normal cows. Therefore, the pattern of alterations in amino acid metabolism in VED cows could be explained by an increased synthesis of glucogenic precursors to promote gluconeogenesis and an increased synthesis of alternative energy substrate precursors to provide energy production.

Vitamin E deficiency may accelerate lipid oxidation. In the VED group, the concentrations of serum ketone bodies like acetone rose. The beta-oxidation of fatty acids in the mitochondria results in the production of acetone [50]. It was hypothesized that VED stimulated the β-oxidation of fatty acids since there was an elevation in the concentrations of acetone. In addition, the level of lipid peroxidation and lipid peroxidation byproducts that are generated is directly proportional to the amount of unsaturated fats [51]. The results of the present study confirmed this, with higher serum concentrations of unsaturated lipids (L7, L8, L9) and MDA in VED cows. The findings provide additional evidence of the more oxidized cell conditions, which can be achieved by an elevation in the oxidation of fatty acids. This is similar to a study in which VED triggered lipid peroxidation [52]. Malondialdehyde is the main byproduct of lipid peroxidation [53]. A rise in MDA values is associated with an increase in oxidative stress and destruction that is controlled by oxidation [54]. In addition, vitamin E is a strong antioxidant, and the decrease in its levels indicates a decline in the TAC, which also indirectly represents the values of oxidative stress non-enzymatic antioxidants [55]. In addition to high levels of MDA, cows with VED also exhibit low levels of TAC. Thus, VED can induce an oxidative stress response in cows.

Vitamin E deficiency can elicit inflammation. Hepatocytes, in response to the harm to the surrounding tissue, will produce acetylated glycoproteins, which will then act as inflammatory mediators [56]. N-acetylated glycoproteins are a type of acute-phase protein that is seen in higher levels during the initial phases of various pathologies, including inflammation, infection, neoplasia, stress, and trauma [57]. In response to cytokines, these "acute phase" acetyl glycoproteins are mostly generated in the liver parenchymal cells [46]. Oxidative stress can occur in cows with VED because of lipid peroxidation. In this investigation, N-acetyl glycoproteins and O-acetyl glycoproteins were found with larger signal strength in the spectra of serum from the VED group contrasted with the normal cows. This may have been in response to oxidative stress, and the VED cows could have had increased liver or peripheral inflammation.

In dairy cattle, hepatic damage and disorders are most likely to occur during the peripartum period because of the substantial shifts in lipid metabolism [58], oxidative stress, and inflammation that can occur during this time [59]. One common method for determining whether there was damage to the liver is to look for elevated levels of the enzyme AST [60]. A low quantity of albumin can impair immunological performance and hinder cellular immune function [61]. Albumin is another important liver function index that is widely recognized as an evaluation tool for the nutritive and inflammation state of the body [62]. In this study, the levels of AST correlated with hepatic injury were increased, and the levels of albumin were markedly lower in cows with VED related to the normal group. Hence, liver injury may occur in VED cows. In addition, a significant reduction in the serum concentrations of HDL was seen in the VED cows. This observation was consistent with a previous study showing that cows with hepatic injury had low HDL concentrations in the blood [63]. Thus, it is reasonable to speculate that cows with VED exhibit hepatic injury. Altogether, hepatic impairment in VED cows relates to the substantial shifts in lipid metabolism, oxidative stress, and inflammation that are generated by VED during early lactation.

Vitamin E deficiency can change rumen microbial metabolism. In dairy production, formate is produced endogenously by rumen methanogens, which use microbial fermentation to create formic acid and hydrogen [64]. Acetate is one of the principal volatile fatty acids produced by ruminal microorganisms through the fermentation of carbohydrates [65]. Dimethylamine is a choline metabolite mediated by microbiota [66]. Taken together, rumen microbiota can manufacture these products. In the present study, serum formate, acetate, and dimethylamine were elevated in the VED group when contrasted with the normal group, which is an interesting finding that VED might alter rumen microbial metabolism, consequently increasing these serum metabolites. However, further studies in rumen microbiota are required to confirm this.

## 5. Conclusions

In this study, a metabolomics framework following ^1^H NMR analysis was used to study metabolic changes in VED cows. We accordingly identified 24 metabolites that showed differential expression between VED and normal cows. Furthermore, 11 metabolic pathways involving energy, carbohydrate, and amino acid metabolism showed significant changes in VED cows. The present findings suggest that VED cows were more likely to experience a negative energy balance characterized by alterations of common systemic metabolic processes and develop oxidative stress, inflammation, and ultimately liver injury. To the best of our knowledge, this is the first research on the metabolic conditions of VED cows observed in vivo. Further studies should use targeted metabolomics to verify metabolite levels and analyze related regulatory enzymes in metabolic pathways of interest.

## Figures and Tables

**Table 1:** Ingredient and chemical composition of the early-lactation TMR diet for 28 multiparous *Holstein* dairy cows.

Ingredient	Content (g/kg DM)
Corn silage	250
Alfalfa hay	120
Oat hay	130
Corn grain, ground	251
Wheat bran	55
Soybean meal	106
Rapeseed meal	25
Cottonseed cake	45
Calcium carbonate	5
Salt	5
Calcium phosphate	3
Mineral and vitamin premix ^1^	5
Chemical composition	
Organic matter	919.3
CP	176.4
Ether extract	31.3
NDF	353.1
ADF	191.0
Non-fiber carbohydrate ^2^	358.7
Ca	8.3
P	4.2

^1^ Contained per kg premix: 20,000 mg Fe, 1600 mg Cu, 8000 mg Mn, 7500 mg Zn, 120 mg I, 20 mg Co, 820,000 IU vitamin A, 300,000 IU vitamin D and 10,000 IU vitamin E. ^2^ Non-fiber carbohydrate, calculated by 1000 − (CP + NDF + Fat + Ash).

**Table 2:** Background characteristics of the vitamin E deficiency (VED) group and normal group.

Parameters	Normal (*n* = 14)	VED (*n* = 14)	*p*-Value
Age	3.16 ± 0.27	3.22 ± 0.13	0.230
Parity	2.22 ± 0.64	2.14 ± 0.55	0.363
BCS	3.29 ± 0.24	3.06 ± 0.27	0.012
Milk yield (kg/d)	35.98 ± 2.54	33.23 ± 2.37	0.003
DMI (kg/d)	15.24 ± 0.34	14.61 ± 0.49	<0.001
Vitamin E (μg/mL)	7.33 ± 1.38	2.59 ± 0.77	<0.001
Vitamin A (μg/mL)	0.19 ± 0.06	0.18 ± 0.05	0.318
Vitamin C (μg/mL)	2.66 ± 0.34	2.45 ± 0.67	0.153
Selenium (μg/mL)	0.05 ± 0.01	0.05 ± 0.01	0.683
Zinc (μg/mL)	1.22 ± 0.26	1.28 ± 0.23	0.262
Copper (μg/mL)	0.98 ± 0.31	0.96 ± 0.24	0.425
Calcium (mmol/L)	2.08 ± 0.69	2.07 ± 0.93	0.487
Phosphorus (mmol/L)	2.72 ± 0.38	2.60 ± 0.29	0.178
Magnesium (mmol/L)	1.54 ± 0.31	1.43 ± 0.46	0.232

VED, vitamin E deficiency; BCS, body condition score; DMI, dry matter intake.

**Table 3:** Comparison of serum biochemical indicators between the VED and normal groups.

Parameters	Normal (*n* = 14)	VED (*n* = 14)	*p*-Value
BHB (mmol/L)	1.02 ± 0.34	1.14 ± 0.39	0.197
NEFA (mmol/L)	0.51 ± 0.18	0.78 ± 0.18	<0.001
Glucose (mmol/L)	5.90 ± 2.59	6.84 ± 2.38	0.163
AST (U/L)	74.06 ± 3.62	95.46 ± 7.10	<0.001
ALT (U/L)	43.60 ± 8.30	42.76 ± 8.86	0.399
Albumin (g/L)	58.01 ± 8.62	46.87 ± 7.69	<0.001
TC (mmol/L)	5.65 ± 1.45	5.98 ± 1.84	0.301
LDL (mmol/L)	3.93 ± 0.94	5.05 ± 0.82	0.001
HDL (mmol/L)	1.51 ± 0.21	1.13 ± 0.22	<0.001
TAC (mmol/L)	0.54 ± 0.17	0.24 ± 0.10	<0.001
SOD (U/mL)	114.30 ± 14.67	122.20 ± 15.32	0.088
GSH-Px (U/mL)	31.65 ± 8.05	29.08 ± 8.49	0.209
Catalase (U/mL)	21.75 ± 1.64	22.57 ± 1.19	0.071
MDA (mmol/L)	9.84 ± 1.51	12.59 ± 2.04	<0.001

BHB, β-hydroxybutyrate; NEFA, non-esterified fatty acids; AST, aspartate aminotransferase; ALT, alanine aminotransferase; TC, total cholesterol; LDL, low-density lipoprotein; HDL, high-density lipoprotein; TAC, total antioxidant capacity; SOD, superoxide dismutase; GSH-Px, glutathione peroxidase; MDA, malondialdehyde.

**Table 4:** Differential serum metabolites of the VED and normal groups screened with ^1^H NMR.

No.	Metabolites	*r* ^a^	Trend ^b^	*p*-Value
1	Acetate	0.7093	↑	0.0001
2	Acetone	0.7099	↑	<0.0001
3	Alanine	0.6812	↑	0.0003
4	Citrate	0.5886	↑	0.0023
5	Creatinine	0.5632	↑	0.0046
6	Dimethylamine	0.5760	↑	0.0016
7	Formate	0.7439	↑	<0.0001
8	Glutamate	0.7495	↑	<0.0001
9	Glutamine	0.8136	↑	<0.0001
10	Glycine	0.6464	↑	0.0015
11	Histidine	0.6720	↑	0.0001
12	Lipid, CH_3_-(CH_2_)n-(LDL)	0.6718	↑	0.0004
13	VLDL, -CH_2_-CH_2_-C=O	0.7018	↑	<0.0001
14	Lipid, -CH_2_-C=O	0.7270	↑	<0.0001
15	Lipid, =CH-CH_2_-CH=	0.6644	↑	0.0005
16	Lipid, -CH=CH-	0.5873	↑	0.0005
17	Lysine	0.7403	↑	<0.0001
18	N-acetyl-glycoprotein	0.5738	↑	0.0034
19	O-acetyl-glycoprotein	0.8071	↑	<0.0001
20	Phenylalanine	0.7010	↑	0.0001
21	Pyruvate	0.7923	↑	<0.0001
22	Valine	0.5915	↑	0.0025
23	α-Glucose	0.7466	↑	<0.0001
24	β-Glucose	0.7968	↑	<0.0001

^a^ *r*: correlation coefficient; ^b^ Trend: change trend compared with normal group. (↑): upregulated.

**Table 5:** The main pathways affected between VED and normal groups.

Main Pathway	Total ^a^	Hits ^b^	Raw *p* ^c^	Holm *p* ^d^	−ln(*p*) ^e^	Impact ^f^
Nitrogen metabolism	9	4	<0.001	<0.001	13.75	0
Aminoacyl-tRNA biosynthesis	64	7	<0.001	<0.001	13.40	0
Alanine, aspartate, and glutamate metabolism	23	4	<0.001	0.005	9.58	0.40
D-Glutamine and D-glutamate metabolism	5	2	0.001	0.083	6.84	1.00
Valine, leucine, and isoleucine biosynthesis	11	2	0.006	0.429	5.18	0.33
Glyoxylate and dicarboxylate metabolism	16	2	0.012	0.896	4.43	0.41
Citrate cycle	20	2	0.018	1	3.99	0.13
Pyruvate metabolism	22	2	0.022	1	3.81	0.24
Glycolysis or gluconeogenesis	26	2	0.030	1	3.49	0.13
Phenylalanine, tyrosine, and tryptophan biosynthesis	4	1	0.043	1	3.16	0.50
Glycine, serine, and threonine metabolism	32	2	0.045	1	3.11	0.29

^a^ Total: the total number of compounds in the pathway; ^b^ Hits: the corresponding number of metabolites in one pathway; ^c^ Raw *p*: the original *p* value measured by the enrichment testing; ^d^ Holm *p*: the *p*-value modified utilizing Holm-Bonferroni approach; ^e^ −ln(*p*): the negative logarithm base e of the *p*-value; ^f^ Impact: the pathway influence value calculated from pathway topology analysis.

## Data Availability

The data presented in this study are available on request from the corresponding author.

## Supplementary Materials

The following supporting information can be downloaded at: https://www.mdpi.com/article/10.3390/ani13182957/s1, Table S1: 1H NMR assignments of major metabolites in serum of cows; Table S2: Model parameters of principal component analysis (PCA); Table S3: Model parameters of orthogonal partial least-squares discriminant analysis (OPLS-DA).
