# Supplementary material for: Serum Metabolic Characterization of Vitamin E Deficiency in Holstein Cows during the Transition Period Based on Proton Nuclear Magnetic Resonance Spectroscopy
Source: Animals (Basel). 2023 Sep 19;13(18):2957. doi: 10.3390/ani13182957 (PMC10525730; doi:10.3390/ani13182957)
Supplement: Supplementary file 1 [file animals-13-02957-s001.zip › animals-2536939-supplementary.pdf]

animals

MDPI

Article

Serum Metabolic Characterization of Vitamin E Deficiency in Holstein Cows during the Transition Period Based on Proton Nuclear Magnetic Resonance Spectroscopy

Table S1. ^{1}H NMR assignments of major metabolites in serum of cows.

No.	Metabolites	δ_H (ppm)[a]
1	3-Hydroxybutyrate	1.18(d)
2	Acetate	1.92(s)
3	Acetone	2.23(s)
4	Alanine	1.48(d), 3.77(q)
5	Choline	3.2(s)
6	Citrate	2.54(d), 2.68(d)
7	Creatinine	3.03(s), 3.04(s), 4.05(s)
8	Dimethylamine	2.71(s)
9	Formate	8.45(s)
10	Glutamate	2.07(m), 2.35(m)
11	Glutamine	2.13(m), 2.45(m)
12	Glycine	3.56(s)
13	Histidine	7.05(s), 7.78(s)
14	Isoleucine	0.94(t), 1(d)
15	Lipid, CH3-(CH2)n-(LDL)	0.84(br)
16	Lipid, CH3-(CH2)n-(VLDL)	0.88(br)
17	Lipid, CH3-(CH2)n-(LDL)	1.27(br)
18	VLDL, -CH2-CH2-C=O	1.57(br)
19	Lipid, -CH2-CH=CH-	2.01(br)
20	Lipid, -CH2-C=O	2.23(br)
21	Lipid, =CH-CH2-CH=	2.76(m)
22	Lipid, -CH=CH-	5.30(br)
23	Lactate	1.33(d), 4.11(q)
24	Leucine	0.96(d), 0.97(d)
25	Lysine	1.50(m), 1.72(m), 1.90(m), 2.5(m), 3.01(m), 3.5(m), 4.5(m)
26	N-acetyl-glycoprotein	2.04(s)
27	O-acetyl-glycoprotein	2.14(s)
28	Phenylalanine	7.33(m), 7.38(m), 7.43(m)
29	Phosphorylcholine/Glycerophosphocholine	3.22(s)
30	Pyruvate	2.37(s)
31	Tyrosine	6.9(d), 7.2(s)
32	Valine	0.99(d), 1.04(d), 3.62(d)
33	α-Glucose	3.42(dd), 3.54(dd), 3.71(t), 3.73(m), 3.84(m), 5.23(d)
34	β-Glucose	3.26(dd), 3.41(t), 3.46(m), 3.49(t), 3.90(dd)

[a]Chemical shift, s, singlet; d, doublet; t, triplet; q, quartet; dd, doublet of doublets; m, multiplet; br, broad resonance.

Table S2. Model parameters of principal component analysis (PCA).

Model[1]	Type[2]	A[3]	N[4]	R^2X (cum)[5]	Q^2 (cum)[6]	Title[7]
M1	PCA-X	4	28	0.606	0.312	VED group – Normal group

[1]The number of the model modeled by the SIMCA software, which corresponds to the result file; [2]The model type of SIMCA, PCA-X means to build a PCA model for the sample; [3]The number of principal components of the model; [4]The number of observations of the model (here is the number of samples); [5]The explanation of the model to the X variable; [6]The predictability of the model; [7]The data object corresponding to the model.

Table S3. Model parameters of orthogonal partial least-squares discriminant analysis (OPLS-DA).

Model[1]	Type[2]	A[3]	N[4]	R^2X (cum)[5]	R^2Y (cum)[6]	Q^2 (cum)[7]	Title[8]
M2	OPLS-DA	1+1+0	28	0.39	0.918	0.792	VED group – Normal group

[1]The number of the model modeled by the SIMCA software, which corresponds to the result file; [2]The model type of SIMCA, OPLS-DA means to build an OPLS-DA model for the sample; [3]The number of principal components of the model; [4]The number of observations of the model (here is the number of samples); [5]The explanation of the model to the X variable; [6]The explanation of the model to the Y variable; [7]The predictability of the model; [8]The data object corresponding to the model.
